# Impact of Polypharmacy, Drug-Related Problems, and Potentially Inappropriate Medications in Geriatric Patients and Its Implications for Bulgaria—Narrative Review and Meta-Analysis

**DOI:** 10.3389/fpubh.2022.743138

**Published:** 2022-03-03

**Authors:** Tzvetan Krustev, Petya Milushewa, Konstantin Tachkov

**Affiliations:** Faculty of Pharmacy, Medical University of Sofia, Sofia, Bulgaria

**Keywords:** drug-related problems, elderly patients, inappropriate prescribing, polypharmacy, meta-analysis, Bulgaria

## Abstract

**Objective:**

Polypharmacy and inappropriate prescribing are overlooked issues in Bulgaria. We aimed at collecting and analyzing global literature on the most prevalent risk factors and investigating what they could reveal about current practice.

**Materials and Methods:**

A systematic narrative review and meta-analysis was conducted on the topic, investigating the prevalence of polypharmacy, odds of potentially inappropriate medications (PIMs) due to polypharmacy, and the likelihood of developing a drug-related problem (DRP) due to PIMs. The results were then related to current demographic statistics to estimate the potential impact on Bulgarian elderly patients.

**Results:**

The prevalence of polypharmacy was estimated at 41% in elderly populations. The odds of a potentially inappropriate medication being prescribed were 2.095, with an expected 30.84% of those leading to a DRP. These numbers indicated that the expected Bulgarian elderly with polypharmacy should be 709,676 with 212,903 cases of DRPs.

**Conclusion:**

Global polypharmacy rates seem to be on the rise, with an expected increase in DRPs.

## Introduction

Polypharmacy refers to the use of multiple medications in a patient, with the most common class of patients utilizing multiple medicines being older adults with multimorbidity. Polypharmacy has seen increased interest, due to the increased consumption of medicines leading to clinical issues (1). Despite multiple definitions of what polypharmacy is—both numerical definitions of the number of drugs, and descriptive definition of polypharmacy, the most commonly used definition is the application of five or more medicines, which is used as the working definition in this article (2). According to the literature, the term may be escalated or de-escalated to include the following three groups, which we have accepted for the article:

a. Excessive polypharmacy (EPP): ten or more different drugs.
b. Polypharmacy (PP): the use of five to nine drugs.
c. No polypharmacy: taking four or fewer drugs (3).

Multimorbidity, characterized by two or more chronic conditions in older adults, has become more pressing and prevalent in light of the aging population coupled with an increase in life expectancy (4). The most common chronic conditions are the following: hypertension, dyslipidemias, ischemic heart disease, diabetes, arthritis, heart failure, depression, chronic kidney disease, osteoporosis, Alzheimer's, COPD, atrial fibrillation, cancer, asthma, and stroke. Multimorbidity is associated with an increased risk of death, low quality of life, disabilities, and adverse drug events (5). It is important to note that multimorbidity and comorbidity are two separate terms. Comorbidity refers to a central disease, with diseases associated with it, whereas multimorbidities can exist independently of each other in a patient (6). Nevertheless, the treatment of a handful of chronic conditions in a single patient necessitates the use of multiple therapeutic agents (7). The prevalence of these multimorbidity diseases is also the main factor in the most-commonly prescribed inappropriate medications, as illustrated by the Beers criteria (8). There are many issues associated with multiple and excessive uses of drugs, which can lead to drug-related problems (DRPs). DRPs also have varying definitions in the literature and can be a broad category, associated with improper use of drugs (9). According to the Pharmaceutical Care Network Europe Association (PCNE), “a DRP is an event or circumstance involving drug therapy that actually or potentially interferes with desired health outcomes,” with the causes varying from drug-related (dose, application, and duration) to patient-related (due to behavior or transfer between primary, secondary, or tertiary care) (10). DRPs are of particular concern in older patients due to the following reasons (11):

- Aging increases the risk of multiple morbidities due to physiological changes, requiring multiple medications.

- Adverse drug reaction (ADR) happens when the patient is taking the normal dosage of the drug and they are more common in the elderly due to metabolic and drug clearance changes. Potentially inappropriate medication (PIM), identified by Beer's and STOPP (Screening Tools of Older Person's potentially inappropriate medications) (12) criteria, refer to drugs that have a high likelihood of causing ADRs. They are frequently used tools to identify PIM.

- Drug interactions: potential drug-drug interactions risk increases with the use of multiple medications. The most frequent suspects in such drug-drug interactions are cardiovascular medicines.

- Prescribing cascade: This occurs when more drugs are prescribed to cope with the adverse drug effect that has developed. Often this is mistaken for a new medical condition.

- Medication non-adherence: polypharmacy brings challenges to medication adherence especially for elder patients with cognitive and/or visual disorders.

- Hip fracture risk: polypharmacy is identified as a risk factor in the elder population.

- Over the counter (OTC) and complementary medication use: such as food supplements not shared with healthcare professionals brings an increased risk of herb-drug interactions for the patient. For example, such an interaction is the use of an angiotensin-converting enzyme (ACE) inhibitor and St. John's wort.

- Transition of care: transitions in care between hospitals and home settings can bring errors in medication. Frequently, the therapy of patients is changed increasing the risk of polypharmacy.

- Pharmacokinetics changes linked to aging: Changes happening in adults in terms of absorption, distribution, metabolism, and elimination of drugs.

- Pharmacodynamics changes associated with aging: organ function changes with aging and response to drugs too. These changes are extremely specific for the different drugs and can vary.

Other factors that influence the risk of DRPs are under-treatment or overtreatment, leading to under-prescribing or overprescribing. Under-treatment can be defined as “prescription of less than the recommended therapy,” whereas overtreatment can be defined as “the intensive treatment of an older adult in whom the harms outweigh the benefits” (13).

As evident, multiple factors can be the reasons for polypharmacy prescriptions, however, these factors vary by patient, with each patient having different odds of developing a DRP. Polypharmacy increases this likelihood and can be the reason for a PIM prescription.

Prevention of polypharmacy and PIM is key in saving costs for the healthcare system, lessening the burden on patients, particularly in older patients (14), and poses a challenge in front of healthcare providers. There are available standardized, evidence-based tools which are used in the practice to assess and reduce ADRs and PIMs in older people. The Beers criteria list is one such tool. Initially published in 1991 as a list of PIMs to be used in older patients, it has evolved to the level of a clinical practice guideline prepared by experts, aimed at avoiding PIMs in adults. Another screening tool for older people prescription is STOPP/START criteria (15). They are similar to the Beers criteria in terms of being formed as a list of drugs that healthcare professionals should stop and, respectively, start prescribing in adults, based on specific situations. These STOPP criteria were arranged according to physiological systems (16). Unlike Beers, STOPP/START criteria can be used as a checklist, which provides a quick way to assess a medication of patients. These types of criteria are medication-centric. Certain authors recommend a patient centered framework, aimed at the reduction of PIM (5). These approaches consist of patient conversations about the goals of therapy, patient concerns, and preferences in conjunction with the aforementioned criteria, to better tailor the therapy to reduce PIM risk.

The impact of polypharmacy and multimorbidity for Eastern Europe, especially Bulgaria, has not been explored in depth. Particular studies have focused on a certain disease area (17), the role of the pharmacist in prevention (18), or the way patient consultation plays a role (19, 20). Any in-depth exploration of the issue requires a systematization of knowledge and practices, which prompted our interest in the topic. The purpose of this review is to systemize the available knowledge on the topic and investigate the prevalence and likelihood of PIMs for this end, a literature review was undertaken and relevant articles were screened and included. An additional goal was to conduct a meta-analysis, investigating the odds of being prescribed an inappropriate medicine with the risk factor being polypharmacy, and the odds of developing a DRP conditional on the fact a PIM is prescribed. Lastly, we wanted to investigate what our results could mean for the Bulgarian reality, based on current demographics and trends.

## Materials and Methods

### Article Selection

Several literature searches were conducted in the Pubmed database with the following keywords:

-Older people AND drug related problems;
-Drug interaction AND older people and inappropriate medication;
-Comorbidity OR concordant disease.

Relevant articles were included if they met the following inclusion criteria:

-Population: Articles with elderly people (defined as 65 years or over).
- Time period between 2000 and 2020.
- Intervention: Either observational (retrospective or prospective) or randomized trials of DRPs or PIMs of polypharmacy vs. no-polypharmacy.
- Outcome: Studies that reported a concrete outcome measure such as Prevalence of DRP or OR of developing a PIM.
-Outcome: Studies that had specified their criteria for a DRP and/or PIM either STOPP/START, Beers, or implicit criteria, which were well-defined and documented.

Studies were divided and analyzed based on the object they were reporting—either DRPs or PIMs. It was also important for the meta-analysis, that these studies had a quantitative measure and reported a specific odds ratio or prevalence of the reported events, and were not summary articles or narrative reviews, which are part of the exclusion criteria.

Exclusion criteria: Systematic reviews, articles of a descriptive nature, studies without a set study population, or outside the predefined time period.

**Figure 1:**
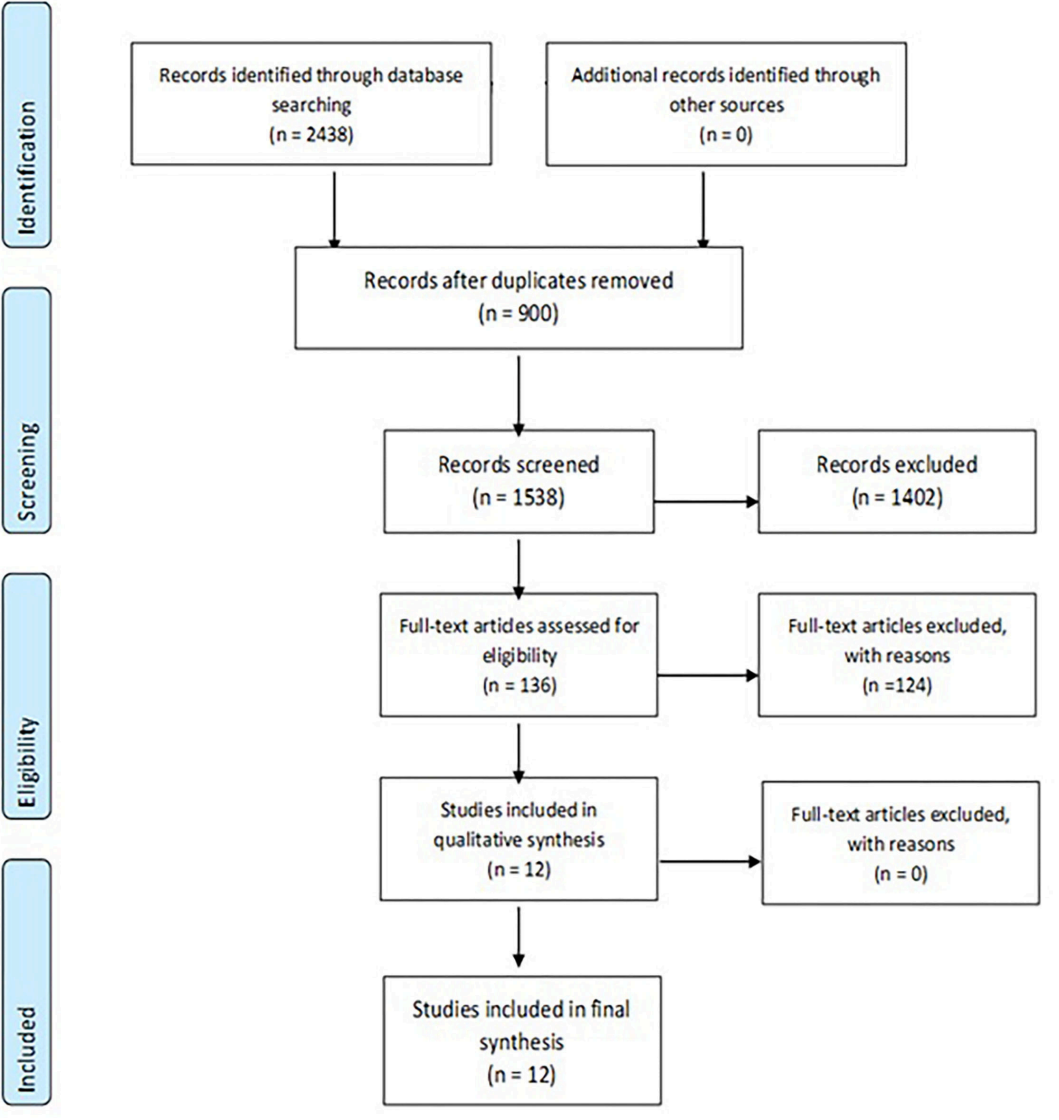
The PRISMA flow diagram of the work process.

A total of 2,438 initial hits were screened by T.K. through their abstract. Abstract reading and removal of duplicates led to 136 relevant articles identified, which were used as the basis for three consecutive full-article reviews. The final outcomes of the performed reviews were 12 publications corresponding to both the inclusion and exclusion criteria—see Table 1 and Figure 1.

**Table 1:** Inclusion and exclusion of articles and work flow.

**Keywords**	**Results**	**Abstracts screened**	**Full article review**	Dropped on first review^*^	Included after second review^**^	Total selected^***^
Comorbidity and concordant disease	453	453	25	21	4	
Drug interactions, older people, inappropriate medication	311	311	18	14	4	
Older people and drug-related problems, PIM	1,674	1,674	93	68	25	12

* *First review was conducted by TK*.

** *Second review by independent party*.

*** *Selected with lead investigators and statistics team*.

### Narrative Review

All the 12 articles were summarized to construct a narrative review. They were broken down to assess the following components:

-Articles reporting the factors, leading to a DRP.
-Articles investigating the link between DRPs and increased hospital admissions.
-Articles investigating the link between hospital admissions, PIM, and polypharmacy.
-Articles investigating the link between PIMs and DRPs.
-Articles reporting on the patient aspect and quality of life (QoL).

### Meta-Analysis

All the calculations were done with MedCalc Version 20.009 by MedCalc Software Ltd., Ostend, Belgium. Studies were separated based on the reporting of PIMs and DRPs and subsequent meta-analyses were initiated to investigate two main questions: what are the odds of being prescribed a PIM if there is polypharmacy, and what is the proportion of PIMs which result in a DRP. From the 12 articles included, only those, which reported the desired outcome were included in the meta-analysis.

## Results

### Included Studies

Outline and description of the included studies are described in Table 2. In total 12 articles were identified matching closely the inclusion criteria for the meta-analysis. The narrative review focuses on these 12 articles to establish the links, investigated in the meta-analysis.

**Table 2:** Included studies and their respective criteria.

**References**	**Study name**	**Study description**
Hartholt et al. (21)	Adverse Drug Reactions Related Hospital Admissions in Persons Aged 60 Years and over, The Netherlands, 1981–2007: Less Rapid Increase, Different Drugs	Secular trend analyses for ADR-related hospital admissions for people with age ≥ 60.
Almodóvar et al. (22)	Associations Between Chronic Disease, Polypharmacy, and Medication-Related Problems Among Medicare Beneficiaries	Retrospective cross-sectional analyses of Medicare beneficiaries aged above 65 received MTM services. A negative binomial regression assessed the relationship between age, sex, and chronic health conditions with MRPs.
Chau et al. (23)	Clinical medication reviews in elderly patients with polypharmacy: a cross-sectional study on drug-related problems in the Netherlands	Cross sectional study based on CMR (clinical medication review) of people with age ≥ 65 with polypharmacy (>5 drugs).
Verdoorn et al. (24)	Effects of a clinical medication review focused on personal goals, quality of life, and health problems in older persons with polypharmacy: a randomized controlled trial (DREAMeR-study)	Randomized clinical trial (RCT), people aged > 70 with polypharmacy (>7 long-term medications) receiving CMR. Difference in HR-QoL and number of health problems in control and intervention groups.
Wawruch et al. (25)	Factors influencing the use of potentially inappropriate medication in older patients in Slovakia	Beers criteria applied to evaluate PIM in older people in Slovakia using multivariate analyses.
Fick et al. (26)	Health Outcomes Associated With Potentially Inappropriate Medication Use in Older Adults	Retrospective cohort study using Beers criteria to evaluate the prevalence of PIM.
Verdoorn et al. (27)	Majority of drug-related problems identified during medication review are not associated with STOPP/START criteria	START/STOPP criteria identify PIM and relation with DRP.
Troncoso-Mariño et al. (28)	Medication-related problems in older people in Catalonia: a real-world data study.	Cross sectional study aiming to determine MRP in elders > 65 of age.
Hohl et al. (29)	Polypharmacy, Adverse Drug-Related Events, and Potential Adverse Drug Interactions in Elderly Patients Presenting to an Emergency Department	Retrospective chart review on 300 randomly selected visits by patients over 65 years.
Reich et al. (30)	Potentially Inappropriate Medication Use in Older Patients in Swiss Managed Care Plans: Prevalence, Determinants and Association with Hospitalization	Beers 2012 and PRISCUS criteria were used to identify PIM.
Primejdie et al. (31)	Potentially inappropriate medications in elderly ambulatory and institutionalized patients: an observational study	Observational study in which START/STOPP and PRISCUS criteria were used to identify PIM.

### Narrative Review

#### Factors, Affecting the Likelihood of a DRP or PIM

Among the reviewed articles, four in total focused on the prevalence and causes of DRPs (22, 32–34). What was interesting was that the reasons for medication problems occurring varied between settings and countries. The study by Almodovar et al. which investigated the US perspective, found that the highest cause of DRPs was due to unsafe medication use with 59% of errors, and 15% due to adherence issues, with the rest being attributed to drug-drug interactions (10%) or overtreatment (13%) or other. These results suggest that patients and the way they are taking their medication might be the prevalent factors in causing adverse events. Contrary to these studies, however, a study by Chau et al. for the Netherlands revealed that medication non-adherence or errors in medication use were responsible for a mere 5.8 and 6.6%, respectively. The bulk of DRP categories was either due to overtreatment (25.5%) or under-treatment (15.9%). Suggesting reasons for these discrepancies would be speculation at this point, but differences between systems are likely the root of this variability.

Primejdie et al. reported that Romanian ambulatory patients under-prescriptions represented 55.34% of PIMs. Over-prescription accounted for 6.9%, and 37.37% of the identified PIMs were due to mis-prescription. The study also investigated institutionalized patients and found that over-prescriptions are responsible for 27.14% of PIMs, mis-prescriptions for 37.73%, and only 10.71% were under-prescribed patients. The most striking difference here is the change in over-prescriptions from ambulatory to the institutionalized patient with a 20-percentage point increase. This is an interesting result because it shows that hospitalizations increase the risk of more drugs being added to a therapeutic regimen, however, this, in turn, increases the risk of a DRP occurring. Troncoso-Marino et al. revealed that commonly found PIMs were those that increase the risk of falling (66.8%), however, contraindicated medicines were also prescribed in both chronic disease (12.1%) and liver disease (4.2%). The study found that among patients, receiving multiple medicines, the prevalence of potentially inappropriate prescriptions was up toward 62.8%.

What these studies reveal is that PIM prevalence is high. Hospitalizations increase the likelihood of polypharmacy, which in turn increases the risk of a DRP occurring, leading sometimes to more hospitalizations, creating a vicious loop.

#### Link Between DRPs and Increased Hospital Admissions

Two studies published results regarding only adverse events as a DRP and hospital admissions (35, 36).

A retrospective study in the Netherlands found that the incidence of ADR hospital admissions increased from 1981 to 2007 by 15 points (from 23.3 to 38.3 per 10,000 persons). Hospitalization due to ADRs has increased in both men and women and according to the results, they have seen a 143% increase in 26 years.

Another study by Hohl et al. showed that ADRs are responsible for 10.6% of all emergency department visits. The authors showed that polypharmacy was related to the development of ADRs with the frequency being 11.5% in patients taking between 2 and 5 medicines and 16.9% in those taking 5 or more medications.

Despite these two studies being easily summarized they nonetheless show a very important link between elderly patients, adverse drug events, and hospitalization rates. Taking into account the four studies, documenting the likelihood of PIMs, a very clear link is illustrated between polypharmacy, inappropriate prescriptions, and hospital admissions.

#### Hospital Admissions, Potentially Inappropriate Medication, and Polypharmacy

As illustrated, patients with polypharmacy can be admitted to hospital due to adverse drug reactions, but one study, aiming to identify risk factors enhancing the probability of use of PIM in hospitalized older patients under the conditions of the Slovak healthcare system, published results about the fact that hospital admission oftentimes leads to additional medication prescriptions (37). According to Wawruch et al. polypharmacy was present in 60.3% of patients at the time of hospital admission and 62.3% at the time of discharge. To identify the use of PIM, the Beers 2003 criteria were applied. At least one PIM was prescribed to 21.0% of all 600 patients included in the study, irrespective of whether this was at the time of hospital admission or discharge. The authors concluded that these findings are consistent with previously published literature and that polypharmacy, immobilization, heart failure, and depression were identified as predictors of the use of PIM.

#### Potentially Inappropriate Medication and DRP Link

Two studies published results regarding the link between PIM and DRP, in particular how ADRs are managed (22, 38). STOPP and START criteria are used to identify potentially inappropriate prescribing and potential prescribing omissions. These criteria provide recommendations on whether a medicine should be initiated or stopped. One of the studies done by Dutch community pharmacists aims to determine to what extent STOPP/START corresponds to DRPs and a summary of the outcomes is the following.

The majority (81%) of DRPs of community-dwelling older patients are not associated with STOPP/START criteria. Despite this, the recommendations to start a medication were higher than those to stop, with 13% START and 5.7% STOPP criteria present in identified DRP. Nonetheless, 65% of DRP identified during medication reviews were not associated with recommendations to cease, replace, or add a drug and cannot be detected with START/STOPP criteria. This seems to suggest that those current tools fall short of addressing the issue with polypharmacy and DRPs.

The other study is observational in which START/STOPP and PRISCUS criteria were used to identify PIM. Results show that ambulatory patients are divided into three groups 37.73%—misprescribed, 55.34%—underprescribed, and 6.92%—overprescribed and, respectively, institutionalized patients are 62.14%—misprescribed, 10.71%—underprescribed, and 27.14%—overprescribed.

The conclusion is that PIM identified in both elderly groups suggested potential risks for the occurrence of adverse events specific to the elderly population.

#### Quality of Life Measurement/Change for Patients on Which Is Performed Clinical Medication Reviews (CMR)

An additional aspect of polypharmacy and DRPs not often explored is the patient perspective. Only one of the studies investigated the health-related quality of life of patients in whom potential risks were addressed before the development of a DRP (33). A randomized controlled trial (RCT) was performed in 35 community pharmacies and cooperating general practices in the Netherlands. Community-dwelling older persons (>70 years) with polypharmacy (>7 long-term medications) were randomly assigned to usual care or to receive a critical medication review (CMR). The primary outcomes were HR-QoL (assessed with EuroQol [EQ]-5D-5L and EQ-visual analog scale [VAS]) and the number of health problems (such as pain or dizziness), after 3 and 6 months. Results show that patients with performed clinical medication reviews had increased quality of life HR-QoL (EQ-VAS): +3.4 p.p. plus reduced number of health problems: −12% and HR-QoL (EQ-5D-5L): −0.022 and reduced health problems: −0.30 vs. the control group. Prevention of DRPs and addressing PIMs improves HR-QoL in addition to preventing further healthcare costs.

### Meta Analyses

#### Polypharmacy and Potentially Inappropriate Prescriptions

**Figure 2:**
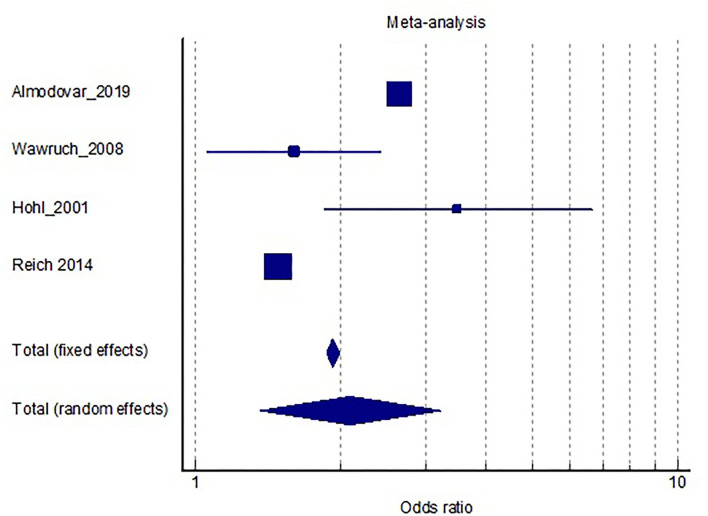
Distribution of results and size of the effect regarding the risk of being prescribed a PIM with polypharmacy as a risk factor.

The prevalence of polypharmacy in the elderly was on average 41% across studies (21, 32, 36, 37). Only two studies analyzed the prevalence of excessive polypharmacy with an average of 14.7%. Four of the analyzed studies reported the relationship between the number of medications prescribed and PIMs. Overall, the fixed effects model showed that patients on 5 or more drugs have a nearly 2 times higher likelihood of having a potentially inappropriate prescription (see Table 3). Studies with many patients enrolled had discrepancies in the odds, which could be due to the different settings, since the study by Almodovar et al. was for the US healthcare system, while the study by Reich et al. encompassed the Swiss national system. It is also worth noting, that the study by Reich et al. presented an aggregate summary of data, which we then extrapolated to the number of patients which could explain the difference in ratios. Nonetheless, in all studies the confidence intervals were above 1, further supporting the argument that polypharmacy is associated with the prescription of PIMs (see Figure 2).

**Table 3:** Results and odds ratios from the meta-analysis.

**References**	**Intervention**	**Controls**	**Odds ratio**	**95% CI**	***z***	***P***
Almodóvar et al. (22)	6,759/14,091	3,521/13,674	2.658	2.527–2.796		
Wawruch et al. (25)	96/374	40/226	1.606	1.063–2.427		
Hohl et al. (29)	48/153	15/130	3.505	1.853–6.629		
Reich et al. (30)	4,204/16,490	6,204/33,178	1.488	1.423–1.556		
Total (fixed effects)	11,107/31,108	9,780/47,208	1.930	1.868–1.995	39.162	<0.001
Total (random effects)	11,107/31,108	9,780/47,208	2.095	1.360–3.226	3.356	0.001

#### Potentially Inappropriate Medication and Risk of Developing a DRP

**Figure 3:**
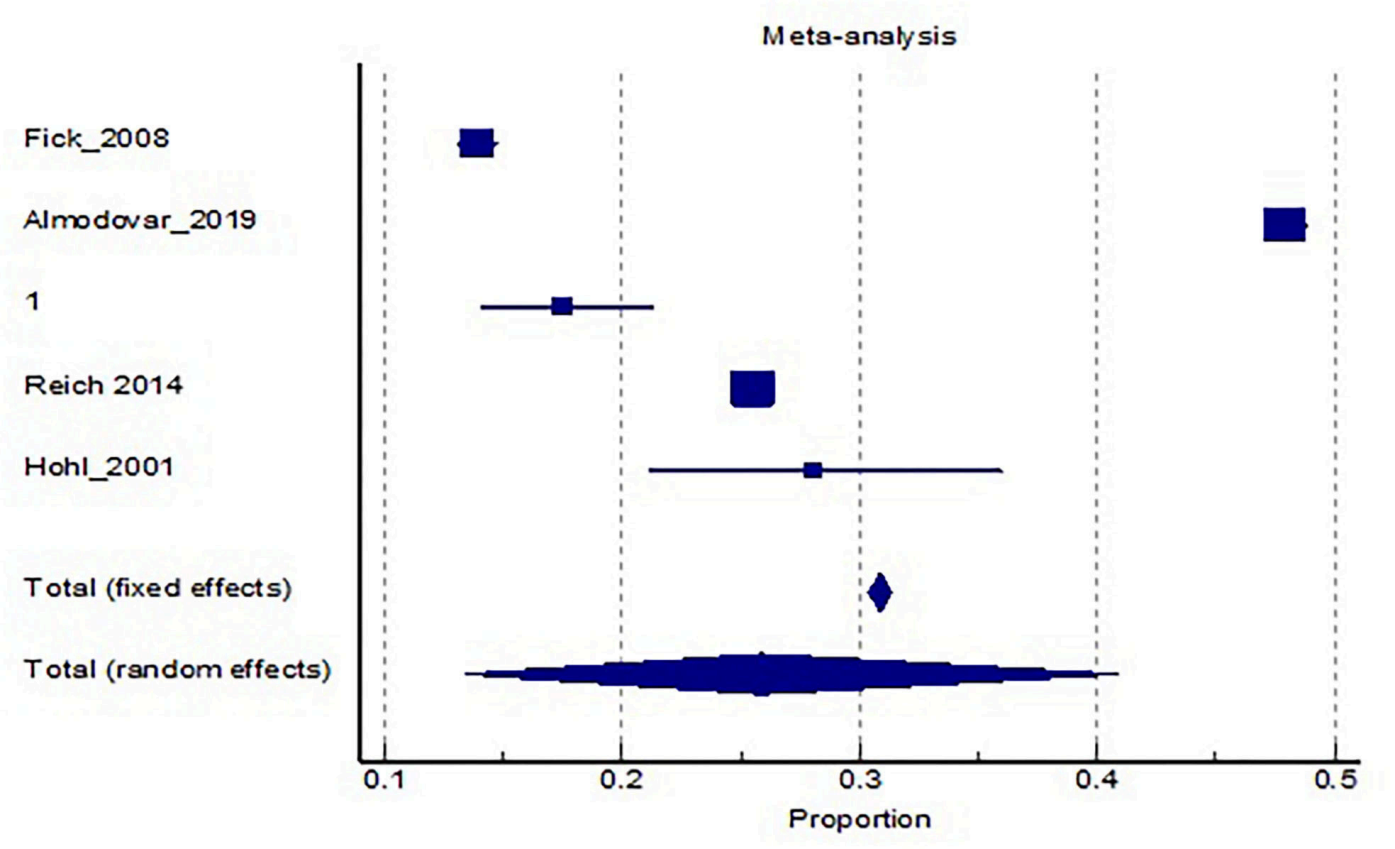
Meta-analysis and distribution of results regarding the risk of developing a DRP with a PIM present.

Given the fact that polypharmacy predisposes to inappropriate prescriptions, we wanted to investigate the likelihood of patients experiencing a side-effect due to an inappropriate prescription. In total, five studies provided information on this link (21, 32, 36, 38, 39). Pooled analysis of studies showed that around 30% of patients will experience a side-effect related to the inappropriate drug. The likelihood seems to be population—dependent, and health system-dependent, whereby the prevalence of DRPs ranged from 13.8 to 47.9%. Two studies investigated the likelihood of a DRP leading to an emergency department visit. Out of the patients, experiencing a side effect, 14.1% will result in a severe case, necessitating a visit to an emergency department (refer to Table 4 and Figure 3).

**Table 4:** Results of proportion distribution and likelihood of a DRP.

**References**	**Sample size**	**Proportion (%)**	**95% CI**
Fick et al. (26)	6,875	13.862	13.053–14.701
Almodóvar et al. (22)	14,091	47.967	47.139–48.796
Verdoorn et al. (27)	457	17.505	14.132–21.306
Reich et al. (30)	16,490	25.494	24.830–26.167
Hohl et al. (29)	153	28.105	21.145–35.933
Total (fixed effects)	38,066	30.848	30.384–31.314
Total (random effects)	38,066	25.895	13.357–40.872

### Implications for Bulgaria

Based on the results from the analyses, we can extrapolate the impact on the Bulgarian elderly population.

**Figure 4:**
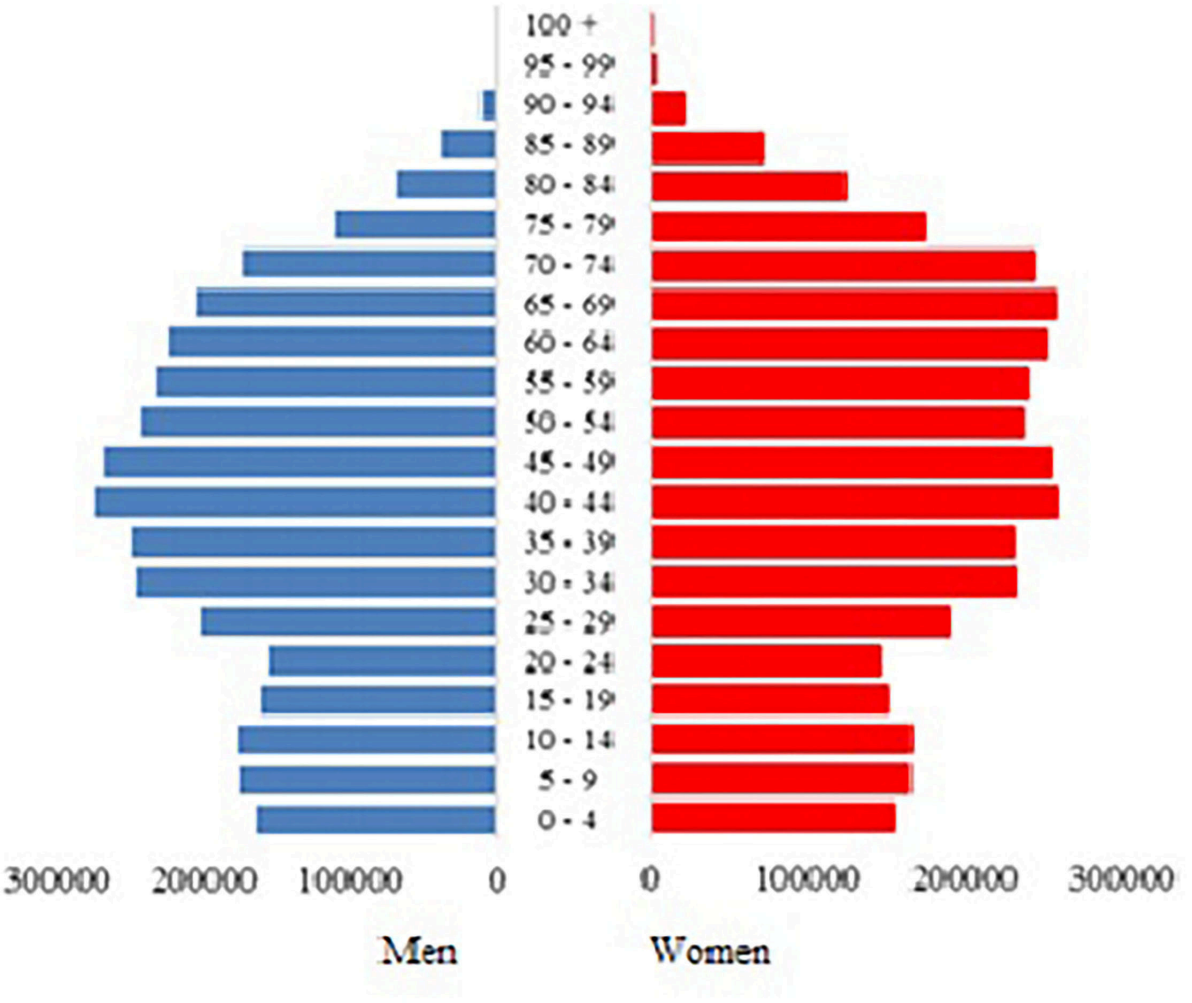
Age distribution of the Bulgarian population.

**Figure 5:**
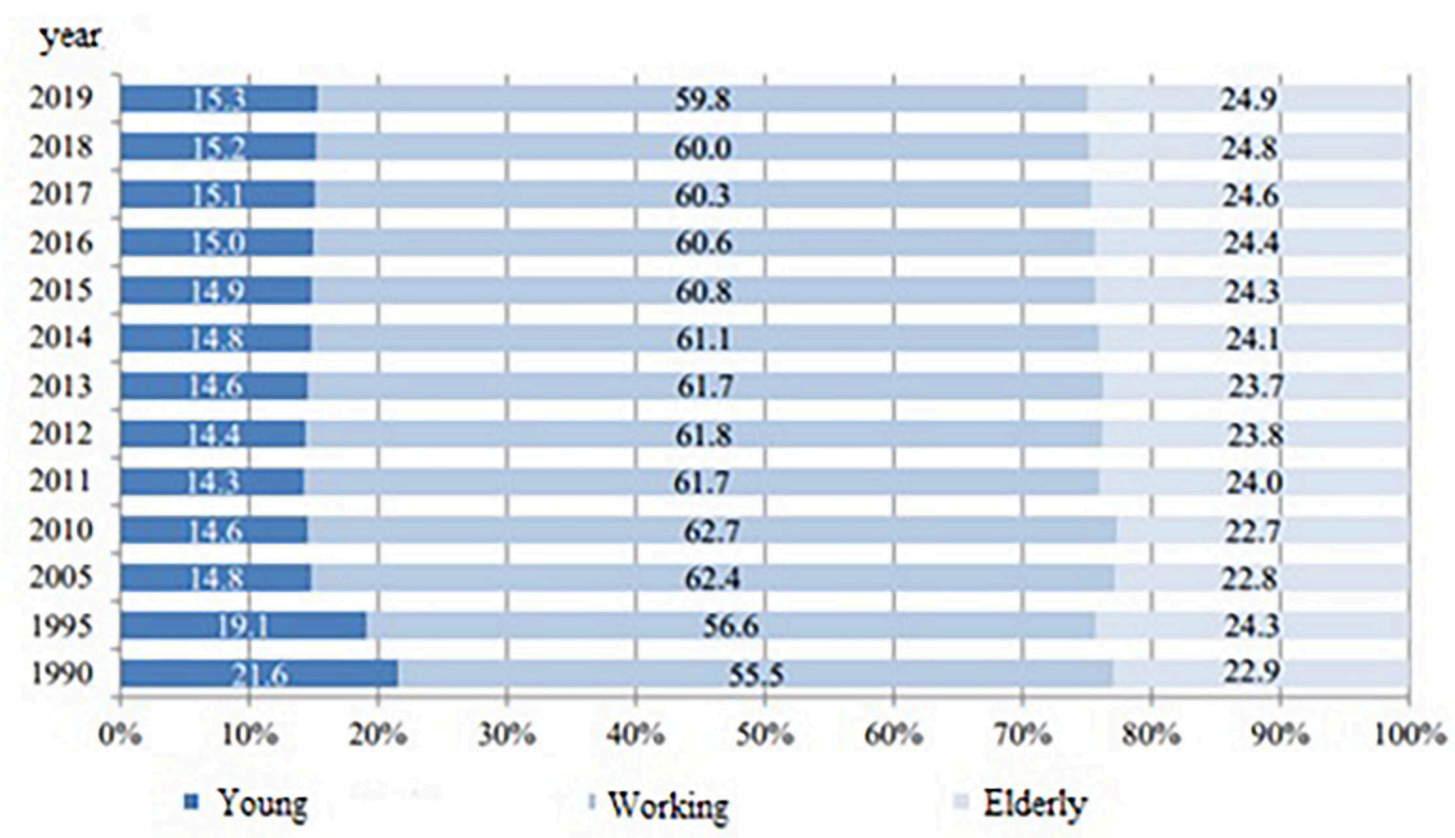
Percentage distribution of the population.

According to the latest data from the National Statistical Institute in Bulgaria, the proportion of elderly people in the country is 24.9%, which translates to 1.73 million people (Figures 4, 5). With a prevalence of 41%, 709,676 elderly citizens are expected to have 5 or more prescribed medication regimens. Taking into consideration that roughly 30% of patients with polypharmacy would experience a medication-related side effect, this leads to an expected 212,903 cases of DRPs in the elderly subgroup of the population, which is a problem currently overlooked by both national practices and legislation.

These figures are disturbing on a few accounts. As of the time of writing of this article no national guidelines are established for any kind of medication review in the elderly. What's more, pharmacy-care services are purely voluntary on the part of both patients and pharmacies. The fact that the rate of DRPs has not been studied in the country reveals a serious gap in knowledge, which could have potential cost-saving implications. Bulgaria is the country in Europe with the highest share of expenditures on medicines and medical devices−36%, with 40% on inpatient care, 18% on outpatient care, and only 3% on prevention (35). In comparison, western European countries spend more on inpatient and outpatient care than on pharmaceuticals. Belgium, Germany, and France spend, respectively, 36, 27, and 32% on inpatient care and 13, 19, and 17% on pharmaceuticals (32, 33, 40). Furthermore, the distribution of costs is not equal in the country. A total of 73% of the expenditures on pharmaceuticals are out-of-pocket expenses, paid by the patient, compared to 27% by health insurance (37). Introducing medication review, with a potential lessening of medication burden and number of medications would positively affect this statistic.

Potentially inappropriate medications (PIMs) and DRPs are problems that should be tackled preemptively, especially for Bulgaria. Official demographic statistics for 2019 show that the proportion of elderly in the country is around 25%, which is already 4% higher than the average for Europe (39). According to a press release from preliminary analysis of the 2021 population census data, confirms these estimates, showing that the Bulgarian population had reached 6.5 million people, out of which 23.9% or 1.5 million people are elderly over the age of 65 (38). Furthermore, the proportion of working-age individuals has shrunk from 65.4% in 2011 to 60.3% in 2021 (38), which would put more pressure on the already strained Bulgarian Health Insurance Fund, particularly in times of a pandemic. Finding ways to reduce costs and improve care for the elderly should be a priority for the country within the next decade. Analysis of PIMs and prevention of DRPs is a potential opportunity to strengthen pharmacist-physician cooperation for the benefit of elderly patients. This would require both local, regional, and national actions, such as improving collection and systematization of DRPs, strengthening the cooperation between family doctors and community pharmacists, and the cooperation within hospitals in the joint pharmacotherapy committees, but also giving them better tools to achieve this—such as improved digital patient records. A recent analysis by Byrne et al. revealed that when such institutions are strengthened, pharmacists' identification of DRPs resulted in recommendations, which were accepted by 89.2% of physicians and 69.4% of consultant physicians (34).

Currently, there are no national guidelines regarding the systematic collection of PIMs, and only adverse drug effects are monitored through the Yellow Card Scheme. Furthermore, the digitalization of the healthcare sector of the country is in its infancy, having begun in earnest in 2017 (36), and is simultaneously implementing stages 1 and 2 of the project. Evidently, there is a long journey to improve patient care, but the opportunities to implement changes in a meaningful way exist, which is why such analyses are important for the national picture and strategy.

Our ambitions are to build upon this work to introduce tangible results, which could serve as the basis of future investigations into actual problems pertaining to the elderly and their care. Our research has several weaknesses. The lack of local data has forced us to rely on international studies and use those results as an approximation to local realities. Despite meta-analyses being satisfactory tools to obtain pooled analysis, analyzing disparate health systems and approaches is bound to introduce an element of bias. Our attempt to limit bias and commit to strict inclusion criteria is a potential remedy, however, this has resulted in much too narrow of a study selection. Nevertheless, our study has revealed a major knowledge gap, which we have tried to rectify.

## Conclusion

Global polypharmacy rates seem to be on the rise, with expected increases in DRPs associated with it. While many countries have introduced criteria to identify potentially inappropriate prescriptions or medication-review standards and the issue is being studied, Bulgaria has yet to investigate the problem fully. This knowledge gap could have cost-saving implications.

The most recent data show that Bulgaria has above average share of elderly patients compared to other European countries, which is why action should be taken preemptively. Strengthening pharmacist-physician cooperation would be of benefit both to patients and the health system.

Currently, the highest share expenditures in healthcare are for medicines and medical devices and are among the highest in the EU, after Cyprus. This is further complicated by the fact that DRPs or PIMs are not investigated in the country and there are no set guidelines on documenting or preventing these two aspects of patient care. The results of this study raise awareness of the issue, particularly on a national level. Physicians and pharmacists should be encouraged to introduce into their practice tools aimed at minimizing inappropriate prescribing and medications, such as the Beers criteria, or to monitor current medication in use, such as the START/STOPP criteria since no national guidelines exist.

Another issue, which is highlighted in this article is the high costs associated with inpatient care and outpatient care. Given the fact that hospital admissions affect the subsequent medication burden after discharge, this should further incentivize healthcare establishments to implement criteria for minimizing the risk associated, particularly in elderly patients, who could have a high medication burden already.

## Data Availability Statement

The original contributions presented in the study are included in the article/supplementary material, further inquiries can be directed to the corresponding author/s.

## Author Contributions

KT and TK: study conception and design and meta-analysis. PM and TK: data collection and draft manuscript preparation. KT, TK, and PM: analysis and interpretation of results. All authors reviewed the results and approved the final version of manuscript.

## Conflict of Interest

The authors declare that the research was conducted in the absence of any commercial or financial relationships that could be construed as a potential conflict of interest.

## Publisher's Note

All claims expressed in this article are solely those of the authors and do not necessarily represent those of their affiliated organizations, or those of the publisher, the editors and the reviewers. Any product that may be evaluated in this article, or claim that may be made by its manufacturer, is not guaranteed or endorsed by the publisher.
